# GRL-09510, a Unique P2-*Crown*-Tetrahydrofuranylurethane -Containing HIV-1 Protease Inhibitor, Maintains Its Favorable Antiviral Activity against Highly-Drug-Resistant HIV-1 Variants *in vitro*

**DOI:** 10.1038/s41598-017-12052-9

**Published:** 2017-09-25

**Authors:** Masayuki Amano, Pedro Miguel Salcedo-Gómez, Ravikiran S. Yedidi, Nicole S. Delino, Hirotomo Nakata, Kalapala Venkateswara Rao, Arun K. Ghosh, Hiroaki Mitsuya

**Affiliations:** 1 Departments of Infectious Diseases and Hematology, Kumamoto University School of Medicine, Kumamoto, 860-8556 Japan; 2 Experimental Retrovirology Section, HIV and AIDS Malignancy Branch, Center for Cancer Research, National Cancer Institute, National Institutes of Health, Bethesda, MD 20892 USA; 3 Department of Biochemistry, Faculty of Medicine, University of Toronto, Toronto, ON Canada; 4 Departments of Chemistry and Medicinal Chemistry, Purdue University, West Lafayette, IN 47907 USA; 5 National Center for Global Health and Medicine Research Institute, Tokyo, 162-8655 Japan

## Abstract

We report that GRL-09510, a novel HIV-1 protease inhibitor (PI) containing a newly-generated P2-*crown*-tetrahydrofuranylurethane (*Crwn*-THF), a P2′-methoxybenzene, and a sulfonamide isostere, is highly active against laboratory and primary clinical HIV-1 isolates (EC_50_: 0.0014–0.0028 μM) with minimal cytotoxicity (CC_50_: 39.0 μM). Similarly, GRL-09510 efficiently blocked the replication of HIV-1_NL4-3_ variants, which were capable of propagating at high-concentrations of atazanavir, lopinavir, and amprenavir (APV). GRL-09510 was also potent against multi-drug-resistant clinical HIV-1 variants and HIV-2_ROD_. Under the selection condition, where HIV-1_NL4-3_ rapidly acquired significant resistance to APV, an integrase inhibitor raltegravir, and a GRL-09510 congener (GRL-09610), no variants highly resistant against GRL-09510 emerged over long-term *in vitro* passage of the virus. Crystallographic analysis demonstrated that the *Crwn*-THF moiety of GRL-09510 forms strong hydrogen-bond-interactions with HIV-1 protease (PR) active-site amino acids and is bulkier with a larger contact surface, making greater van der Waals contacts with PR than the *bis*-THF moiety of darunavir. The present data demonstrate that GRL-09510 has favorable features for treating patients infected with wild-type and/or multi-drug-resistant HIV-1 variants, that the newly generated P2-*Crwn*-THF moiety confers highly desirable anti-HIV-1 potency. The use of the novel *Crwn*-THF moiety sheds lights in the design of novel PIs.

## Introduction

Currently available combination antiretroviral therapy (cART) has had a significant impact on human immunodeficiency virus type-1 (HIV-1) infection and acquired immunodeficiency syndrome (AIDS). Recent analyses have revealed that mortality rates for HIV-1-infected patients have become close to that of general population^1–4^. Moreover, an increase in the number of patients receiving cART has brought about more than 35% decline in the number of newly infected individuals in developing countries including Sub-Saharan nations^5^. However, 36.7 million individuals were living with HIV-1 infection in 2015, and only limited number of HIV-1-infected individuals received cART^5^. Furthermore, the eradication of HIV-1 continues to be elusive, due to the viral reservoirs persisting in various tissues including lymph nodes and the central nervous system (CNS)^6–8^. The emergence of drug-resistant HIV-1 variants, long-term cART-induced toxicities, the inability to fully restore normal immunologic functions once AIDS developed, development of various HIV-1-infection-assocaited cancers, and HIV-1-associated neurocognitive disorders (HAND) also exacerbate the limitations of the current cART.

**Figure 1:**
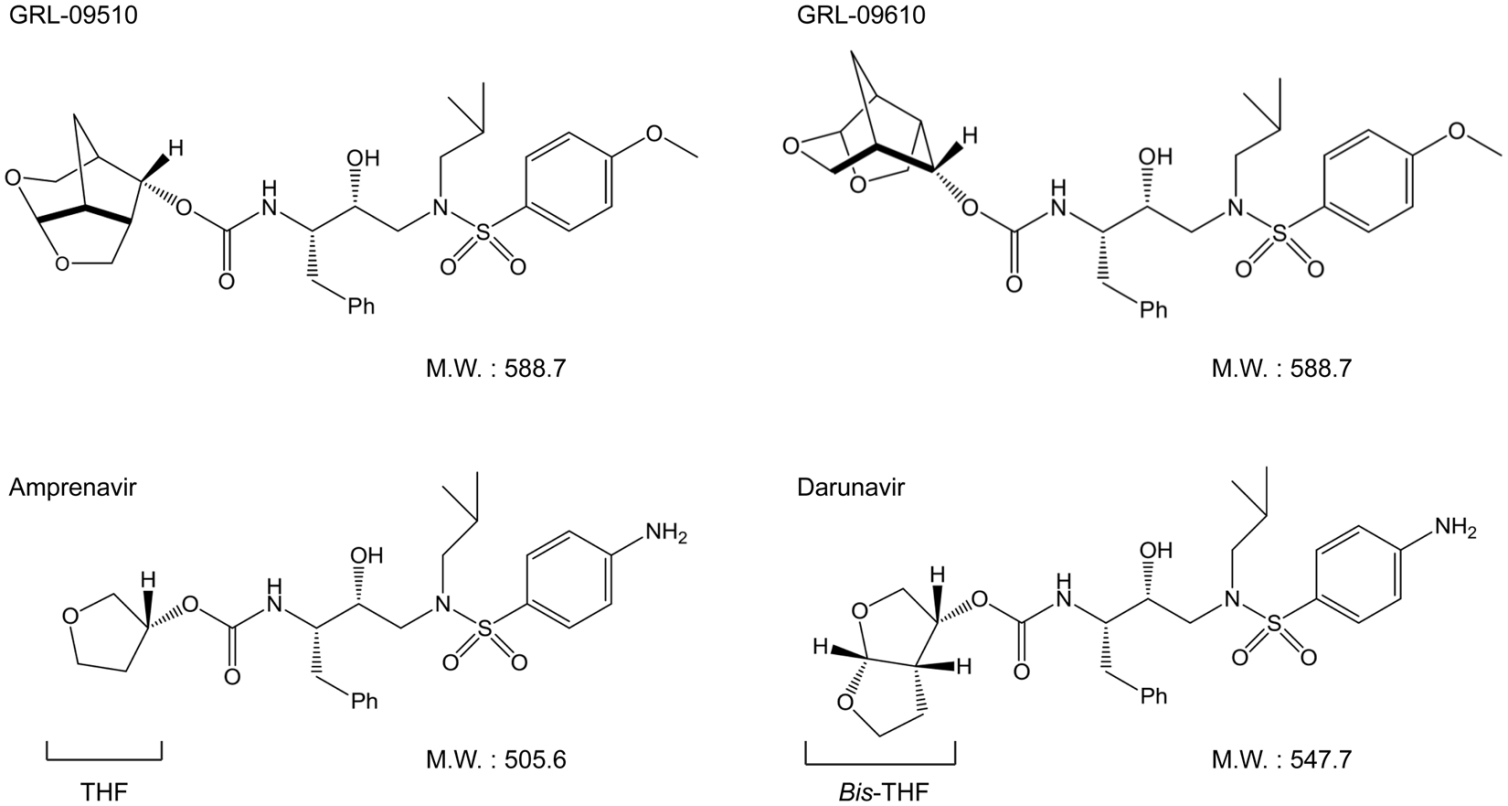
Structures of GRL-09510, GRL-09610, amprenavir, and darunavir.

Although the recent first-line cART with boosted PI-based and integrase inhibitor-based regimens has made the development of drug-resistant HIV-1 relatively less likely over an extended period of time^9,10^, various limitations of cART listed above are still contributing for the emergence of HIV-1’s drug-resistance^11–16^. We have been focusing on the design and synthesis of non-peptidyl PIs that are active against HIV-1 variants highly resistant to the currently approved PIs. In the present work, we designed, synthesized, and identified two novel PIs, GRL-09510 and -09610, which contain a unique polycyclic *crown*-THF (*Crwn*-THF) as the P2 moiety and a sulfonamide isostere (Fig. 1). We found that GRL-09510 exerts strong activity against a wide spectrum of laboratory HIV-1 strains, an HIV-2 strain, and primary clinical isolates including highly-multi-drug-resistant HIV-1 variants with minimal cytotoxicity. We also carried out the selection experiments of GRL-09510-resistant HIV-1 variants by propagating a laboratory wild-type HIV-1_NL4-3_ strain in the presence of increasing concentrations of the compound, and determined the amino acids substitutions that emerged under the pressure of GRL-09510 in the PR or Gag-encoding region. We, furthermore, performed crystallographic analyses to determine how GRL-09510 interacts with the active-site amino acids of PR.

## Results

### Antiviral activity of GRL-09510 against HIV-1_LAI_ and HIV-2_ROD_

We first examined the antiviral potency of GRL-09510 against a wild-type HIV-1. The activity of GRL-09510 against HIV-1_LAI_ was greater with an EC_50_ (50% effective concentration) value of 0.0014 µM compared to other clinically available PIs examined including DRV (Table 1), as assessed with the MTT assay using MT-2 target cells. GRL-09510 was also active against HIV-2_ROD_ with an EC_50_ value of 0.0018 µM (Table 1). GRL-09510’s cytotoxic profiles were favorable with CC_50_ (50% cytotoxic concentration) being 39.0 µM and the selectivity indices proved to be 27,857 and 21,667 for HIV-1_LAI_ and HIV-2_ROD_, respectively (Table 1). The activity of GRL-09610, an isomer of GRL-09510, against HIV-1_LAI_ was comparable to that of GRL-09510; however, GRL-09610’s activity against HIV-2_ROD_ was compromised as seen in the activity of APV, ATV, LPV, and DRV.

**Table 1:** Antiviral activity of GRL-09510, -09610 against HIV-1_LAI_, HIV-2_ROD_ and cytotoxicity against MT-2 cells.

Compound	EC_50_ (μM)		CC_50_ (μM)	^*^Selectivity Index (CC_50_/EC_50_)	
	Against HIV-1_LAI_	Against HIV-2_ROD_		MT2/HIV-1_LAI_	MT2/HIV-2_ROD_
GRL-09510	0.0014 ± 0.0009	0.0018 ± 0.0001	39.0 ± 0.7	27,857	21,667
GRL-09610	0.0023 ± 0.0007	0.015 ± 0.009	33.1 ± 1.0	14,391	2,207
APV	0.037 ± 0.007	0.297 ± 0.174	48.2 ± 9.9	1,303	162
ATV	0.004 ± 0.001	0.017 ± 0.003	32.4 ± 1.0	8,100	1,906
LPV	0.023 ± 0.003	0.021 ± 0.007	26.7 ± 4.2	1,161	1,271
DRV	0.005 ± 0.002	0.014 ± 0.007	100.6 ± 8.8	20,120	7,186

MT-2 cells (10^4^/ml) were exposed to 100 TCID_50_ of HIV-1_LAI_ or HIV-2_ROD_ and cultured in the presence of various concentrations of each PI, and the EC_50_ values were determined by the MTT assay. All assays were conducted in duplicate, and the data shown represent mean values derived from the results of two or three independent experiments. *Each selectivity index denotes a ratio of 50% cytotoxicity (CC_50_) to EC_50_ against HIV-1_LAI_ or HIV-2_ROD_.

### GRL-09510 exerts strong activity against highly PI-resistant clinical HIV-1 isolates

In our previous work, we isolated four highly multi-PI-resistant primary HIV-1 strains (HIV-1_MDR/B_, HIV-1_MDR/C_, HIV-1_MDR/G_, HIV-1_MDR/TM_) from patients with AIDS, who had failed then-existing cART regimens after receiving 9 to 10 anti-HIV-1 drugs over 34 to 83 months^17,18^. These primary strains contained 11 to 15 amino acid substitutions in the PR-encoding region, which have been reportedly associated with HIV-1 resistance against various PIs (see Supplemental Table 1). The four different multi-drug resistant clinical isolates (HIV_MDR_) used in the assays shown in Table 2 contain various resistance-associated amino acid mutations in the reverse transcriptase (RT) as well as in the PR. All four patients from whom these variants were isolated had received 6 different NRTIs and 1 of the four had received 1 NNRTI. The activity of APV, ATV, and LPV against such HIV_MDR_ strains was significantly compromised as examined in PHA-PBMC as target cells using p24 production inhibition as an endpoint (Table 2). However, GRL-09510 exerted strong antiviral activity with its EC_50_ values against those HIV_MDR_ ranging 0.0032~0.0040 μM, the same range of the activity of GRL-09510 against HIV-1_ERS104pre_, a wild-type HIV-1 strain isolated from a drug-naïve individual (Table 2). The antiviral activity of GRL-09510 proved to be the most effective against those HIV_MDR_s examined compared to the four Food and Drug Administration (FDA)-approved PIs (APV, ATV, LPV, and DRV). We also examined antiviral activity of GRL-09510 against a highly DRV-resistant variant (HIV-1_DRV_^R^_20P_)^19^. This variant was generated using the mixture of 8 highly multi-PI-resistant clinical isolates as a starting HIV-1 source and selected with increasing concentrations of DRV. GRL-09510 maintained its activity against HIV-1_DRV_^R^_20P_ (EC_50_: 0.025 μM), being more effective than DRV by 12-fold (Table 2). In addition, we determined antiviral activity of GRL-09510 against an ×4-tropic subtype-A strain, R5-tropic subtype-B strain, and a dual tropic subtype-B strain in PHA-PBMCs. As can be seen in Supplemental Table 2, GRL-09510 effectively inhibited the replication of all of the strains employed.

**Table 2:** Antiviral activity of GRL-09510 and -09610 against multi-drug resistant clinical isolates in PHA-PBMCs.

Virus^a^	EC_50_ (μM)^b^					
	GRL-09510	GRL-09610	APV	ATV	LPV	DRV
HIV-1_ERS104pre_ (wild-type)	0.0035 ± 0.0001	0.0032 ± 0.0002	0.039 ± 0.005	0.0031 ± 0.0006	0.028 ± 0.002	0.0044 ± 0.0009
HIV-1_MDR/B_	0.0037 ± 0.0001 (1)	0.0145 ± 0.0013 (5)	0.42 ± 0.05 (11)	0.36 ± 0.16 (116)	>1 (>36)	0.019 ± 0.002 (4)
HIV-1_MDR/C_	0.0032 ± 0.0006 (1)	0.0049 ± 0.0018 (2)	0.25 ± 0.05 (6)	0.05 ± 0.02 (16)	0.39 ± 0.05 (14)	0.007 ± 0.003 (2)
HIV-1_MDR/G_	0.0033 ± 0.0014 (1)	0.0158 ± 0.0133 (5)	0.30 ± 0.09 (8)	0.03 ± 0.01 (10)	0.18 ± 0.02 (6)	0.020 ± 0.009 (5)
HIV-1_MDR/TM_	0.0040 ± 0.0006 (1)	0.0289 ± 0.0016 (9)	0.32 ± 0.05 (8)	0.071 ± 0.008 (23)	0.55 ± 0.09 (20)	0.028 ± 0.007 (6)
HIV-1_MDRmix DRV_^R^_20P_	0.025 ± 0.001 (7)	0.25 ± 0.03 (78)	>1 (>26)	>1 (>323)	>1 (>36)	0.30 ± 0.06 (68)

^a^HIV-1_ERS104pre_ served as a source of wild-type HIV-1. Amino acids sequence of each variant was described in Supplemental Table 1. ^b^The EC_50_ (50% effective concentration) values were determined by using PHA-PBMC as target cells and the inhibition of p24 Gag protein production by each drug was used as an endpoint. The numbers in parentheses represent the fold changes of EC_50_ values for each isolate compared to the EC_50_ values for HIV-1_ERS104pre_. All assays were conducted in duplicate or triplicate, and the data shown represent mean values (±1S.D.) derived from the results of two to four independent experiments. PHA-PBMCs were derived from a single donor in each independent experiment.

### GRL-09510 is active against various PI-selected laboratory HIV-1 variants

We also examined GRL-09510 against an array of HIV-1_NL4-3_ variants, which had been selected by propagating HIV-1_NL4-3_ as a starting strain, in the presence of increasing concentrations (up to 5 µM) of each of 3 FDA-approved PIs (ATV, LPV and APV) in MT-4 cells^20,21^. Such variants had acquired major HIV-1 PI- resistance-associated amino acid substitutions in their PR-encoding region of the viral genome (see Supplementary Table 1). Each variant was highly resistant to its corresponding PI and showed significant resistance with the EC_50_ value of >1 µM. GRL-09510 maintained its antiviral activity against all the variants with EC_50_ values of 0.0037~0.0048 μM (fold-differences were 1~2 compared to against HIV-1_NL4-3_) (Table 3). Overall, GRL-09510 generally exerted significantly favorable antiviral activity against various wild-type HIV-1 strains, drug-resistance variants, and HIV-2 strain than other conventional PIs examined (Tables 1–3). The antiviral activity of GRL-09610 was also found compromised to all the PI-resistant variants employed in the present study as compared to that of GRL-09510 (Tables 2 and 3).

**Table 3:** Antiviral activity of GRL-09510 and -09610 against laboratory highly conventional-PI-resistant variants.

Virus^a^	EC_50_ (μM)^b^					
	GRL-09510	GRL-09610	APV	ATV	LPV	DRV
HIV-1_NL4-3_	0.0028 ± 0.0007	0.0032 ± 0.0002	0.030 ± 0.006	0.0043 ± 0.0009	0.042 ± 0.004	0.0045 ± 0.0004
HIV-1_ATV_^R^_5μM_	0.0037 ± 0.0009 (1)	0.0354 ± 0.0008 (11)	0.35 ± 0.07 (9)	>1 (>233)	>1 (>24)	0.024 ± 0.06 (5)
HIV-1_LPV_^R^_5μM_	0.0039 ± 0.0005 (1)	0.032 ± 0.001 (10)	>1 (>25)	0.038 ± 0.001 (9)	>1 (>24)	0.034 ± 0.005 (8)
HIV-1_APV_^R^_5μM_	0.0048 ± 0.0001 (2)	0.31 ± 0.09 (97)	>1 (>25)	0.371 ± 0.008 (86)	0.40 ± 0.01 (10)	0.41 ± 0.01 (93)

^a^Amino acids sequence of each variant was described in Supplemental Table 1. ^b^The EC_50_ (50% effective concentration) values were determined by using MT-4 cells as target cells. MT-4 cells (10^5^/ml) were exposed to 100 TCID_50_s of each HIV-1, and the inhibition of p24 Gag protein production by each drug was used as an endpoint. All assays were conducted in duplicate or triplicate, and the data shown represent mean values (±1S.D.) derived from the results of two to four independent experiments.

### *In vitro* selection of HIV-1 variants resistant to GRL-09510

**Figure 2:**
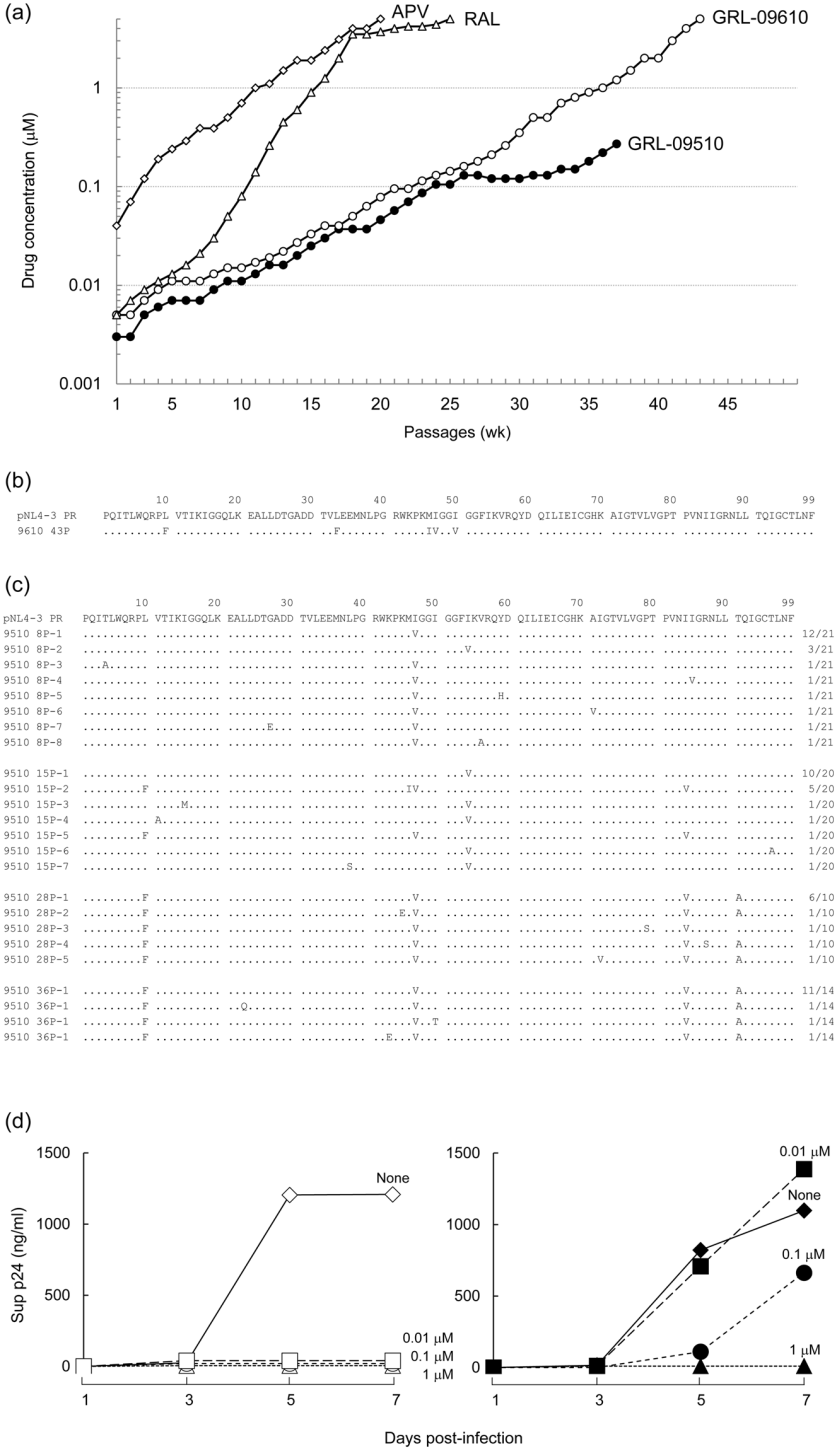
Amino acid sequences of the PR-encoding region of HIV-1 variants selected in the presence of GRL-09510 *in vitro*, and replication kinetics of HIV_9510_^R^_P36_. (**a**) HIV-1_NL4-3_ was propagated in the presence of increasing concentrations of amprenavir (◊) raltegravir (Δ), GRL-09610 (○) or GRL-09510 (●) with a cell-free manner. Selection data of amprenavir-resistant variant was previously reported by us^25^. The p24 concentrations of GRL-09510 selection culture supernatants in passages 8, 15, 28, or 36, were 564, 398, 268, or 265 ng/ml, respectively. (**b**) The amino acid (AA) sequences of protease, deduced from the nucleotide sequence of the protease-encoding region of proviral DNA isolated at passage 43 of GRL-09610’s selection, is shown. The AA sequence of the wild-type HIV-1_NL4-3_ protease is illustrated at the top as a reference. (**c**) The amino acid (AA) sequences of protease, deduced from the nucleotide sequence of the protease-encoding region of each proviral DNA isolated at each indicated time of GRL-09510’s selection, are shown. The number of each clone identified over the number of all the clones generated and sequenced is shown on the far right. (**d**) MT-4 cells were exposed to HIV-1_NL4-3_ (on the left panel) or HIV-1_9510_^R^_P36_ (on the right panel) and each cultured with or without GRL-09510 (the final concentration of MT-4 cells 10^4^/ml, and drug concentrations 0, 0.01, 0.1, or 1 μM). The amount of p24 in each culture flask was measured every two days. HIV-1_9510_^R^_P36_ contains L10F, I47V, I84V, and T90A substitutions in PR region, H87Q and R143K in Gag Capsid (p24) region, and L1F in Gag p6 region.

We next attempted to select HIV-1 variants resistant to GRL-09510 by propagating HIV-1_NL4-3_ in MT-4 cells in the presence of increasing concentrations of GRL-09510 as previously described^22–24^. As shown in Fig. 2a, HIV-1_NL4-3_ almost immediately started to replicate in the presence of APV and RAL and the selection concentration reached at 5 µM at passage 20 and beyond. HIV-1_NL4-3_ was also selected in the presence of GRL-09610, the isomer of GRL-09510. As shown in Fig. 2a, HIV-1_NL4-3_ started to replicate at around passage 30 and the selection concentration of GRL-09610 reached 5 µM at around passage 43. Under the same condition, HIV-1_NL4-3_ was first exposed to 0.003 µM GRL-09510 and underwent 24 passages for selection concentration to reach a 35-fold greater concentration (0.105 µM) than the initial concentration. By around passage 27, the amounts of p24 Gag protein produced in the culture medium was rather modest (up to ~265 ng/ml); however, at around passage 30, at which the virus began continuously failing to propagate in concentrations >0.27 µM and we discontinued the selection at passage 37. Taken together, the emergence of GRL-09510-resistant variants was significantly delayed, strongly suggesting that GRL-09510 has a substantially high genetic barrier to the emergence of resistant HIV-1 variants.

The PR-encoding region of the proviral DNA isolated from infected MT-4 cells was isolated, sequenced at passage 43 under 5 µM GRL-09610 selection, cloned and sequenced at passages 8, 15, 28, and 36 under GRL-09510 selection. The sequences of the region cloned and the % frequency of identical sequences at each passage are depicted in Fig. 2c. HIV-1_NL4-3_ at passage 43 (5 µM of GRL-09610) had 5 amino acid substitutions in its PR region; L10F, L33F, M46I, I47V, I50V (Fig. 2b), and 4 substitutions in Gag region; H87Q, R229K, V230I in the Capsid-encoding region, and L1F in p6 region. It was thought probable that most of these amino acid substitutions were associated to HIV-1_NL4-3_’s acquisition of resistance to GRL-09610. We then examined GRL-09510-exposed HIV-1_NL4-3_’s acquisition of amino acid substitutions in its PR- and gag-encoding regions in more details. By passage 8, I47V substitution had been acquired in 18 of 21 clones. At passage 15, I54V was seen in 14 of 20 clones and I47V, L10F and I84V were seen in 6 of 20 clones. However, at passage 28, I54V was found to have mutated back to the wild-type and all clones had L10F, I47V, and I84V. At passage 28 and beyond, most clones had T91, which is rarely identified in other PI-resistant HIV-1 variants. By passage 36, all clones had acquired L10F, I47V, I84V, and T91A substitutions in the PR-encoding region (Fig. 2c), H87Q, R143K in its Capsid (p24)-encoding region, and L1F in its p6-encoding region. The locations of all of these amino acid substitutions seen in HIV-1_9510_^R^_36P_ in its PR are illustrated in Supplemental Fig. 1. It was suggested the presence of these amino acid substitutions might relate to the acquisition of viral resistance to GRL-09510. Thus, we further examined the effects of each of the 4 substitutions, by generating single substitution-carrying HIV-1 variants (HIV-1_L10F_, HIV-1_I47V_, HIV-1_I84V_, and HIV-1_T91A_). Each single substitution-carrying HIV-1 variant showed no significant changes in their susceptibility to GRL-09510 (≤2-fold increase), while the fold-difference of the activity of GRL-09510 against HIV-1_9510_^R^_36P_ was 11 as compared to that against HIV-1_NL4-3_ (Table 4). These data, taken together, show that the amino acid substitutions might have made HIV-1 resistant to GRL-09510; however, the level of the resistance endowed seemed to be only modest.

**Table 4:** GRL-09510’s activity against infectious HIV-1 clones carrying each of 4 amino acid substitutions that emerged during the selection with GRL-09510.

Virus^a^	EC_50_ (μM)^b^
	GRL-09510
HIV-1_NL4-3_	0.0022 ± 0.0009
HIV-1_9510_^R^_36P_	0.024 ± 0.002 (11)
HIV-1_L10F_	0.0044 ± 0.0002 (2)
HIV-1_I47V_	0.0036 ± 0.0005 (2)
HIV-1_I84V_	0.0033 ± 0.0003 (2)
HIV-1_T91A_	0.0033 ± 0.0004 (2)

^a^HIV-1_L10F_, HIV-1_I47V_, HIV-1_I84V_, and HIV-1_T91A_ were created using HIV-1_NL4-3_ plasmid clone. ^b^The EC_50_ (50% effective concentration) values were determined by MT-4 cells employing p24 assay. The data shown represent mean values (±1S.D.) derived from the results of four to six independent experiments. The numbers in parentheses represent the fold changes of EC_50_ values compared to the value against HIV-1_NL4-3_.

### HIV-1_9510_^R^_P36_ could grow with a high concentration of GRL-09510

Next, we determined the growth kinetics of HIV-1_NL4-3_ and HIV-1_9510_^R^_P36_ in the presence or absence of GRL-09510. As shown in Fig. 2d, HIV-1_NL4-3_ failed to grow in the presence of as low as 0.01 µM GRL-09510 during the entire culture period of 7 days. However, HIV-1_9510_^R^_P36_ propagated in the presence of 0.01 and 0.1 µM GRL-09510 and production of significant amounts of p24 was seen in the culture medium over the 7-day culture period (Fig. 2d) although the replication of HIV-1_9510_^R^_P36_ was completely suppressed in the presence of 1 µM GRL-09510, indicating that HIV-1_9510_^R^_P36_ did not acquire high levels of resistance to GRL-09510. We further asked whether HIV-1_9510_^R^_P36_ propagated in the presence of various PIs including GRL-09510. The fold-differences in the EC_50_ values of all FDA-approved PIs examined against HIV-1_9510_^R^_P36_ was only 2 to 9 as compared to their EC_50_ values against HIV-1_NL4-3_. The fold-difference of the EC_50_ value of GRL-09510 against HIV-1_9510_^R^_P36_ was also moderate (11-fold). These data also indicate that the resistance level acquired in HIV-1_9510_^R^_P36_ against various PIs was moderate (Supplemental Table 3).

### X-ray crystallographic analysis of PR_WT_ in complex with GRL-09510

**Figure 3:**
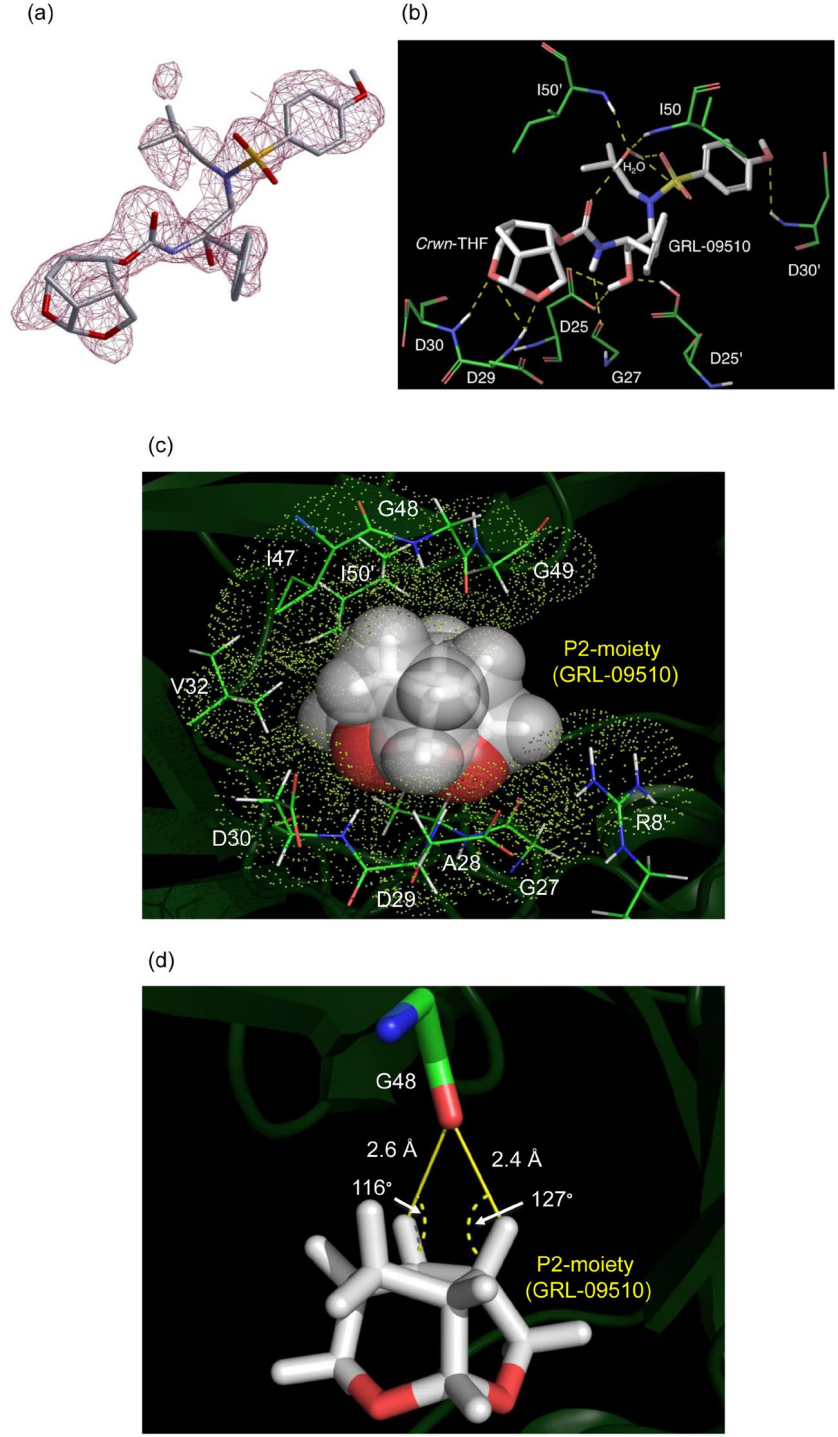
The hydrogen bonding profile of GRL-09510, and Van der Waals contacts of P2-moiety of GRL-09510 with PR_WT_. (**a**) The difference electron density map for GRL-09510 is shown. GRL-09510 is shown in stick representation with carbon, nitrogen, oxygen and sulfur atoms in grey, blue, red and yellow colors, respectively. The type of difference electron density map is |Fo|-|Fc| and is contoured at 2.5 σ. (**b**) GRL-09510 shows multiple H-bonds in the active site of PR_WT_. Water is shown as red sphere and the polar contacts are shown as yellow dashed lines. The carbons of PR and GRL-09510 are shown in green and white colors, respectively. Oxygens, nitrogens, and sulfurs are shown in red, blue, and yellow, respectively. PR residues are labeled as 1 to 99 and 1′ to 99′ for monomers 1 and 2 of the PR dimer, respectively (PDB ID: 5V4Y). (**c**) The P2-moiety of GRL-09510 (spheres) shows multiple Van der Waals (VdW) contacts with PR_WT_. The PR residues are shown as thin sticks with VdW surface in dots. The carbons of PR and GRL-09510 are shown in green and white colors, respectively. Oxygen atoms, nitrogen atoms, and hydrogen atoms are shown in red, blue, and white, respectively. (**d**) The P2-moiety of GRL-09510 (white sticks representation) shows two potential C—H---O contacts with G48 (green sticks representation) in the S2 binding pocket of PR_WT_. Oxygen and nitrogen atoms are color coded by red and blue, respectively.

The X-ray crystal structure of PR_WT_ in complex with GRL-09510 was solved in the space group *P*6_1_22 with one PR monomer per asymmetric unit. GRL-09510 was found to bind in two alternate orientations (separated by 180°) to the active site of PR_WT_ dimer as evident from the difference-electron density map shown in Fig. 3a. In order to analyze the hydrogen bonds (H-bonds), hydrogen atoms were added and their orientations were optimized sampling the crystallographic water molecules through the protein preparation wizard in Maestro (v9.0 Schrodinger LLC). As shown in Fig. 3b, the P2-moiety of GRL-09510 forms three strong H-bonds with the backbone amide hydrogen atoms of D29 (two H-bonds with inter-atomic distances: 1.8 Å and 2.9 Å) and D30 (one H-bond with inter-atomic distance: 2.2 Å). One strong H-bond was seen with the backbone carbonyl oxygen atom of G27 with an inter-atomic distance of 2.6 Å. The transition-state-mimic hydroxyl group of GRL-09510 showed one H-bond each with the side chain δ-oxygen atoms of D25 and D25′ with inter-atomic distances of 2.2 Å and 1.5 Å, respectively. The P2′-methoxybenzene moiety of GRL-09510 forms one strong H-bond with the backbone amide hydrogen atom of D30′ with an inter-atomic distance of 2.7 Å. One conserved crystallographic water molecule was seen bridging between GRL-09510 and the backbone amide hydrogen atoms of I50 and I50′ (inter-atomic distances ranging between 1.5 and 2.0 Å). GRL-09510 had multiple VdW contacts with the active site residues of the PR. Especially, the P2-moiety showed enhanced VdW contacts in the S2-binding pocket as seen with darunavir (DRV)^25^. As shown in Fig. 3c, the P2-moiety completely filled the S2-binding pocket of the PR with multiple VdW contacts. The P2-moiety of GRL-09510 shows multiple VdW contacts with residues G27, A28, D29, D30, V32, I47, G48, G49, R8′ and I50′. Two hydrogen atoms from the P2-moiety of GRL-09510 pointing towards the backbone carbonyl oxygen atom of G48 were found to be within the interatomic distances of 2.4 Å and 2.6 Å, indicating the formation of strong CH---O bonds (Fig. 3d).

## Discussion

GRL-09510, which contains a newly-generated polycyclic non-peptide P2-*crown*-tetrahydrofuranylurethane (*Crwn*-THF) and a sulfonamide isostere, potently suppressed the replication of wild-type HIV-1 and HIV-2 with favorable EC_50_ values. GRL-09510 maintained its potent antiviral activity against a variety of clinical HIV_MDR_ isolates with EC_50_ values ranging from 0.0032 to 0.0040 μM, while the existing FDA-approved HIV-1 PIs examined either failed to suppress the replication of those isolates or required much higher concentrations to inhibit their replication. GRL-09510 also potently inhibited the replication of laboratory-selected HIV-1 PIs-resistant variants with similarly low EC_50_ values.

Of note, we have previously demonstrated a few prototypic PIs (Supplemental Fig. 2) that were highly potent against two wild-type HIV-1s (HIV-1_NL4-3_ and HIV-1_WT/ERS104pre_) and a multi-drug resistant clinical strain (HIV-1_MDR/G_)(Supplemental Table 4) with EC_50_ values ranging from 0.5 to 20 nM. However, compared to GRL-09510 that has an EC_50_ value of as low as 3.3 nM against an HIV-1 variant (HIV-1_APV_^R^_5µM_) resistant to APV that structurally resembles DRV, all these prototypic PIs were much less potent against HIV-1_APV_^R^_5µM_ with EC_50_ values ranging from 38 to 560 nM. Moreover, GRL-09510 exerted potent activity against a highly DRV-resistant HIV-1 variant (HIV-1_MDRmixDRV_^R^_20P_)^19^ with an EC_50_ value of 25 nM (only 7-fold difference to its EC_50_ against a wild-type HIV-1)(Table 2); however, one (GRL-0519) of those prototypic PIs and DRV lost their activity against HIV-1_MDRmixDRV_^R^_20P_ by 50- and 68-fold, respectively (Table 2 and Supplemental Table 4).

Although *in vitro* cell culture may not be much reflective of viral selective pressure imposed by PIs seen in clinical settings, our HIV-1_NL4-3_-based selection experiment using GRL-09510 showed that the emergence of GRL-09510-resistant variants was clearly delayed compared to that of APV- or widely-used integrase inhibitor RAL-resistant variants. APV-resistant HIV-1 variants have been reported to contain V32I, I50V, I54L/M, L76V, I84V, and L90M substitutions at their PR region^26,27^. However, except for I84V, none of such APV-resistance-associated amino acid substitutions were identified during the GRL-09510′s selection (Fig. 2a,c). V11I, V32I, L33F, I47V, I50V, I54M, I54L, T74P, L76V, I84V and L89V are known as DRV-resistance-associated substitutions. Among such substitutions, only two substitutions, I47V and I84V, were seen in our selection experiment using GRL-09510 (Fig. 2c). When we generated and examined HIV-1_I47V_ and HIV-1_I84V_, both variants were as sensitive to GRL-09510 as HIV-1_NL4-3_ (Table 4). These data strongly suggest that the combination of these two substitutions is associated with the observed moderate level resistance to PIs of HIV-1_9510_^R^_P36_.

In our previous studies, selection experiments with certain potent PIs such as TMC-126 and GRL-1398^28^ containing a P2′ *para*-methoxy moiety led to the selection of resistant variants containing a unique A28S substitution in their PR region^17,26^. It is of particular note that the A28S amino acid substitution did not emerge in the present selection experiment with GRL-09510. Intriguingly, when HIV-1_NL4-3_ was selected with GRL-0519, which contains the same P2′ *para*-methoxy group, the A28S substitution did not emerge over 37 passages^20^. GRL-0519 has *tris*-tetrahydrofuranylurethane (*tris*-THF) moiety as the P2 ligand and the *para*-methoxy moiety at the P2′ site, suggesting that the presence of *tris*-THF prevented the emergence of the A28S substitution. In this regard, the combination of the novel P2 *Crwn*-THF and P2′ *para*-methoxy in GRL-09510 appears to have prevented the selection of A28S substitution.

The structural analysis using the X-ray crystallographic data of PR complexed with GRL-09510 showed that the P2-*Crwn*-THF of GRL-09510 forms a strong hydrogen-bond network with the backbone atoms of Asp 29 and Asp 30. In addition, as illustrated in Fig. 3c, the *Crwn*-THF forms greater VdW and hydrophobic contacts with multiple amino acids in the active site of PR than the *bis*-THF moiety of DRV. Such greater VdW contacts of GRL-09510 should be at least in part responsible for GRL-09510’s more potent anti-HIV-1 activity compared with that of DRV. The two strong CH–O bonds between the *Crwn*-THF and the backbone carbonyl oxygen atom of G48 with the interatomic distances of 2.4 Å and 2.6 Å (Fig. 3d) should also be responsible for the observed potent activity of GRL-09510 against HIV-1_WT_ and various drug-resistant HIV-1 variants.

The C_β_, C_γ_ and C_δ_ atoms of Ile47 (located in the highly flexible flap domain of the protease) are engaged in multiple Van der Waals interactions with the P2-*Crwn*-THF moiety of GRL-09510 (Fig. 3c). The I47V substitution (mutation of a longer side chain of Ile to a shorter side chain of Val) results in loss of Van der Waals interactions with P2-*Crwn*-THF due to the absence of C_δ_ atom in Val (HIV-1_9510_^R^_8P_, Fig. 2c). Nevertheless, as shown in Table 4, the HIV-1_I47V_ did not display resistance against GRL-09510, suggesting that the P2-*Crwn*-THF moiety of GRL-09510 still restrains the flap domain from major conformational changes that could affect the inhibitor binding. The P2-*mono*-THF (of APV) or P2-*bis*-THF (of DRV) due to smaller size compared to P2-*Crwn*-THF may not retain their contacts with Val47 generated by the I47V substitution. This confirms that the increase in size favors enhanced binding of GRL-09510 compared to APV or DRV. As shown in Table 4, the substitution T91A failed to select resistant strains of HIV-1 against GRL-09510. However, in the presence of I47V, L10F and I84V substitutions, the T91A substitution (HIV-1_9510_^R^_36P_, Fig. 2c) displayed a 11-fold increase in EC_50_. Thr91 is located in the helical domain of the protease (Supplemental Fig. 1), adjacent to Trp6′, and is engaged in multiple polar contacts with Asn88, Arg87 (directly) and Asp29 (indirectly). The T91A substitution may induce structural distortion in the helix generating a conformational “ripple effect” that may destabilize the H-bonds between P2-*Crwn*-THF moiety of GRL-09510 and the backbone of Asp29. Furthermore, this structural distortion in the helix domain could be worsened by the enhanced Van der Waals interactions between Ala91 (generated by the T91A substitution) and Trp6′. Due to increase in size, GRL-09510 can tolerate both I47V and T91A without any resistance from HIV-1. Thus, the substitutions I47V and T91A require L10F and I84V to cause increased resistance against GRL-09510. Thus, the P2-*Crwn*-THF moiety of GRL-09510 perhaps not only contributes to the enthalpic component of binding via H-bonds with Asp29 and Asp30 (as seen with DRV) but also contributes to the entropic component via Van der Waals interactions with Ile47 as well as Val47 (I47V) due to its increased size.

The P2-*bis*-THF moiety of DRV and P2-moieties of the analogs generated based on DRV were structurally found to be within the chemical space of GRL-09510’s P2-*Crwn*-THF moiety. The two fused-ring structures enforce H-bonding with the backbone of both Asp29 and Asp30. However, the P2-*mono*-THF moiety of APV primarily targets the backbone of Asp30 to favor Van der Waals interactions with Val32 and Ile47. According to the substrate-envelope model proposed by Chellappan *et al*.^29^, APV, DRV and DRV-analogs (including GRL-09510) are within the substrate-envelope of the wild type protease. But the combination of V32I and I47V substitutions confers resistance against APV, which may not necessarily indicate that the P2-*mono*-THF moiety of APV is out of the substrate-envelope. Previously, it has been reported by Yedidi *et al*.^30^, that the accumulation of multiple resistance-associated amino acid substitution leads to an expanded active site cavity of protease. Liu *et al*.^31^ confirmed that such protease variants have an altered and expanded substrate-envelope. The expanded substrate-envelope, due to increase in the chemical space, generates higher penalty on the entropic component of the inhibitor binding but not for substrate binding due to substrate co-evolution. The P2-*Crwn*-THF moiety of GRL-09510 due to its increase in size as well as improved entropy would be a better inhibitor to probe any of changes in the size of substrate-envelope especially in the S2-binding pocket. In this context, further improvement in the size of the P2′-moiety of GRL-09510 may significantly reduce the selection of resistance mutations by filling the S2′-binding pocket as well.

The present data demonstrate that GRL-09510 has favorable features as a candidate drug for treating patients living with HIV-1_WT_ and/or HIV-1_MDR_, and that the newly introduced *Crwn*-THF with *para*-methoxybenzene should be critical for the strong binding of GRL-09510 to PR and should serve as a promising pharmacophore for designing novel PIs. Of note, boosted DRV (DRV/r) monotherapy recently conducted in order to reduce toxicities and costs of the current cART demonstrated that DRV/r monotherapy appears to be a slightly higher risk of serum HIV-1 RNA elevations compared to 2 NRTI plus DRV/r regimens^32^. In this regard, GRL-09510, which has stronger and more favorable antiviral profiles than DRV, may serve as a potential candidate for PI monotherapy.

## Methods

### Cells and viruses

MT-2 and MT-4 cells were grown in RPMI-1640 culture medium with 10% FCS (JRH Biosciences, Lenexa, MD), 50 unit/ml penicillin, and 100 μg/ml kanamycin. The following HIV-1s were employed for the drug susceptibility assay: HIV-1_LAI_, HIV-1_NL4-3_, HIV-2_ROD_, HIV-1_ERS104pre_^33^, a clinical HIV-1 strains isolated from drug-naive AIDS patients, and HIV-1s which were originally isolated from AIDS patients who had received 9–10 anti-HIV-1 drugs over the 34–83 months and were genotypically/phenotypically characterized as multi-PI-resistant HIV-1 variants^17,18^. Amino acid (AA) substitutions in the PR region of PI-resistant strains compared to the consensus type B sequence (Los Alamos database), or wild-type HIV-1_NL4-3_, were summarized at Supplemental Table 1.

### Antiviral agents

Two novel PIs, GRL-09510 and GRL-09610, were designed and synthesized by AKG *et al*. Both compounds were confirmed to be enantiomerically pure. The detailed synthetic methods for the two compounds will be reported elsewhere. Hoffmann-La Roche AG (Basel, Switzerland) kindly provided saquinavir (SQV). Amprenavir (APV) was a gift from GlaxoSmithKline, Research Triangle Park, NC. Lopinavir (LPV) was kindly provided by Japan Energy, Tokyo. Atazanavir (ATV) was a contribution from Bristol-Myers Squibb (New York, NY). Darunavir (DRV) was synthesized as previously described^34^. Raltegravir (RAL) was kindly provided by Dr. Kenji Maeda (NCI/NIH, Bethesda, MD). HIV-1_ME46_ and HIV-1_92UG029_ were provided by NIH/AIDS-Reagent Program.

### Drug susceptibility assays

The susceptibility of HIV-1_LAI_ or HIV-2_ROD_ to various compounds was determined as previously described^20–22^. Briefly, MT-2 cells (10^4^/ml) were exposed to 100 TCID_50_ of HIV-1_LAI_ or HIV-2_ROD_ in the presence or absence of various concentrations of compounds in 96-well plates and were incubated at 37 °C for 7 days. After incubation, 100 μl of the medium was removed from each well, 3-(4,5-dimetylthiazol-2-yl)-2,5-diphenyltetrazolium bromide (MTT) solution (10 μl, 7.5 mg/ml in PBS) was added to each well, followed by incubation at 37 °C for 2 h. To dissolve the formazan crystals, which were generated from MTT by cellular oxidoreductase in living cell, 100 μl of acidified isopropanol containing 4% Triton X was added to each well and the optical density measured in a microplate reader (Vmax; Molecular Devices, Sunnyvale, CA). To determine the sensitivity of clinically isolated HIV-1s to compounds, PHA-PBMC (10^6^/ml) were exposed to 50 TCID_50_ of each HIV-1 and cultured in the presence or absence of various concentrations of compounds in 10-fold serial dilutions in 96-well plates. In determining the drug-susceptibility of certain laboratory HIV-1 strains, MT-4 cells were employed as target cells as previously described^23,24^. MT-4 cells (10^5^/ml) were exposed to 100 TCID_50_ of drug-resistant HIV-1s in the presence or absence of various concentrations of compounds and were incubated at 37 °C. On day 7, the supernatants were harvested and the amounts of p24 were determined by using a fully-automated chemiluminescent enzyme immunoassay system (Lumipulse *f*: Fujirebio, Tokyo)^35–37^. Drug concentrations that suppressed the production of p24 by 50% (EC_50_; 50% effective concentration) were determined by comparison with the p24 production in drug-free control well. All assays were performed in duplicate, and EC_50_ shown in this report represent average values of two to four independent experiments. PHA-PBMCs were derived from a single donor in each independent experiment. The research protocols described in the present study were carried out in accordance with relevant guidelines and regulations, and were approved in Ethics Committee for Epidemiological and General Research at the Faculty of Life Sciences, Kumamoto University.

### *In vitro* selection of PI-resistant HIV-1 variants

MT-4 cells (10^5^/ml) were exposed to HIV-1_NL4-3_ (500 TCID_50_) and cultured in the presence of compounds at an initial concentration of its EC_50_ value. Viral replication was monitored by the determination of the amount of p24 produced by MT-4 cells. The supernatants were harvested on day 7 and were used to infect fresh MT-4 cells for the next round of culture in the presence of increasing concentrations of each compound. Proviral DNAs obtained from the lysates of infected cells were subjected to sequencing. This selection procedure was carried out until the drug concentration reached 5 μM as previously described^20–22^).

### Determination of nucleotide sequences

Molecular cloning and determination of the nucleotide sequences of HIV-1 strains passaged in the presence of anti-HIV-1 agents were performed as previously described^21^. In brief, DNA was extracted from HIV-1-infected MT-4 cells by using the InstaGene Matrix (Bio-Rad, Hercules, CA) and was subjected to cloning, followed by sequence determination. The 1^st^-round PCR mixture consisted of 1 μl proviral DNA solution, 10 μl Ex-*Taq* (Takara Bio Inc., Otsu, Japan), and 10 pmol each of the 1^st^ PCR primers in a total volume of 20 μl. The 1^st^ PCR products (1 μl) were used directly in the 2^nd^-round PCR, and 2^nd^ PCR products were purified with spin-columns (MicroSpin S-400; Amersham Biosciences., Piscataway, NJ), cloned directly using pGEM-T Easy vector (Promega, Fitchburg, WI), and subjected to sequencing with a 3130 automated DNA sequencer (Applied Biosystems, Foster City, CA). The detailed PCR conditions used have been summarized in Supplemental Table 5.

### Determination of viral growth kinetics of GRL-09510-resistant HIV-1_NL4-3_ variants and wild-type HIV-1_NL4-3_

The GRL-09510-resistant variant at passage 36 was propagated in fresh MT-4 cells without GRL-09510 for 7 days, and aliquoted HIV-1_9510_^R^_P36_ viral stocks were stored at −80 °C. MT-4 cells (3.2 × 10^5^) were exposed to the HIV-1_9510_^R^_P36_ or wild-type HIV-1_NL4-3_ preparation containing 10 ng/ml p24 in 6-well plates for 3 hours, and the infected MT-4 cells were washed with fresh medium, divided into 4 fractions and each cultured with or without GRL-09510 (final concentration of MT-4 cells 10^4^/ml, and drug concentrations 0, 0.01, 0.1 and 1 μΜ). The amounts of p24 were measured every two days for up to 7 days.

### Generation of recombinant HIV-1 clones

To generate HIV-1 clones carrying the desired AA substitutions, site-directed mutagenesis was performed with a QuikChange site-directed mutagenesis kit (Stratagene, La Jolla, CA), and the AA substitution-containing genomic fragments were introduced into pHIV-1_NL4-3Sma_, which had been created to have a SmaI site by changing two nucleotides (2590 and 2593) of pHIV-1_NL4-3_.

### Expression, purification and refolding of PR_WT_

Expression/purification/refolding of PR_WT_ were performed as described previously^38^. Briefly, the inclusion bodies isolated from *E.coli* containing PR_WT_ were extracted with 3 M guanidine HCl (GnCl), centrifuged and the supernatant was loaded on Sephadex-200 column that was pre-equilibrated with 4 M GnCl. PR containing fractions were pooled and were further purified by reverse phase column. Fractions were analyzed by SDS-PAGE and the purity was determined to be > 95%. Lyophilized PR_WT_ was dissolved in 1 ml of 50% acetic acid solution and was added drop-wise to 29 ml of refolding buffer (50 mM sodium acetate, pH 5.2, 5% ethylene glycol, 10% glycerol, 5 mM dithiothreitol (DTT), and a 2-fold molar excess of GRL-09510) while stirring on ice. Refolding was continued at 4 °C with constant stirring overnight. The refolded PR_WT_-GRL-09510 complex was concentrated using Amicon filters (3 kDa molecular weight cut off) by centrifugation at 4,800 g. The final PR concentration was determined to be ~2 mg/ml.

### Crystallization of PR-GRL-09510 complexes

Hanging drop vapor diffusion method was used for co-crystallization. PR_WT_-GRL-09510 complex (4 μl) was mixed with 4μl of well solution per drop. Grid screens such as ammonium sulfate, sodium chloride and quick screen (Hampton Research, CA) were used to obtain preliminary crystallization hits. Co-crystals of PR_WT_-GRL-09510 were obtained within one day at room temperature. Clusters of rod shaped PR_WT_-GRL-09510 co-crystals were obtained using 0.8 M sodium/potassium phosphate buffer at pH6.9. The clusters were carefully dissociated using micro tools and individual crystals were picked up into nylon loops. Glucose (30%) was used as cryo-protectant for all the co-crystals. Cryo-coated co-crystals were instantaneously frozen in liquid-nitrogen.

### X-ray diffraction data collection and processing

X-ray diffraction data for PR_WT_-GRL-09510 were collected at the SER-CAT (southeast regional collaborative access team), beam line 22-ID (wavelength: 1.0 Å) at the Advanced Photon Source, Argonne National Labs, IL. A Rayonix MX300HS detector was used to record the diffraction data at a distance of 225 mm from the crystal. The exposure time for each frame was 1 s with a frame width of 0.5°. Diffraction data were processed and scaled using HKL2000^39^. Processing details are given in Supplemental Table 6.

### Structure solution and refinement

Structure solution was obtained using Molecular replacement (MR) as described previously^38^. Briefly, MR was performed using MOLREP^40^ through CCP4^41,42^ interface with PR_WT_ taken from PDB ID:4HLA as a search model. Structure solution was directly refined using REFMAC5^43^. The initial coordinates for GRL-09510 were prepared by modifying the structure of the PI, TMC-126 taken from the crystal structure, PDB ID:2I4U. GRL-09510 was fit into the electron density using ARP/wARP Ligands^44,45^. Initial refinement libraries for GRL-09510 were obtained from REFMAC. Solvent molecules were built using ARP/wARP solvent building module. After building water molecules, the final model was refined using the simulated annealing method from phenix.refine^46^ on the NIH-Biowulf Linux cluster. The root mean square deviation in bond lengths and bond angles significantly improved by geometry-optimized libraries for ligands using the semi-empirical quantum mechanical method of refinement, eLBOW-AM1^47^ during refinement in phenix.refine. Details are given in Supplemental Table 6.

### Structural analysis

The final refined structures were used for structural analysis. Hydrogen bonds were calculated between the heavy atom and the hydrogen atom by using a distance cutoff values of 3.0 Å measured between the donor and acceptor heavy atoms^48^. Cutoff values for angles were minimum donor: 90° and minimum acceptor: 60°. Hydrogen bonds with a distance of >3.0 Å were considered weak interactions. Vander Waals contacts were calculated between two atoms (one from GRL-09510 and one from PR_WT_) with a maximum 3.5 Å distance cutoff.

## Electronic supplementary material

Supplemental Tables 1–6, Supplemental Figures 1,2
