# Supplementary material for: GRL-09510, a Unique P2-Crown-Tetrahydrofuranylurethane -Containing HIV-1 Protease Inhibitor, Maintains Its Favorable Antiviral Activity against Highly-Drug-Resistant HIV-1 Variants in vitro
Source: Sci Rep. 2017 Sep 25;7:12235. doi: 10.1038/s41598-017-12052-9 (PMC5613016; doi:10.1038/s41598-017-12052-9)
Supplement: Supplementary file 1 — Supplemental Tables 1–6, Supplemental Figures 1,2 [file 41598_2017_12052_MOESM1_ESM.pdf]

Supplementary information

GRL-09510, A UNIQUE P2-*CROWN*-TETRAHYDROFURANYLURETHANE -CONTAINING HIV-1 PROTEASE INHIBITOR, MAINTAINS ITS FAVORABLE ANTIVIRAL ACTIVITY AGAINST HIGHLY-DRUG-RESISTANT HIV-1 VARIANTS *IN VITRO*

Masayuki Amano[1], Pedro Miguel Salcedo-Gómez[1], Ravikiran S. Yedidi[2,3], Nicole S. Delino[2], Hirotomo Nakata[1], Kalapala Venkateswara Rao[4], Arun K. Ghosh[4], and Hiroaki Mitsuya[1,2,5]*

[1]Departments of Infectious Diseases and Hematology, Kumamoto University School of Medicine, Kumamoto 860-8556, Japan; [2]Experimental Retrovirology Section, HIV and AIDS Malignancy Branch, Center for Cancer Research, National Cancer Institute, National Institutes of Health, Bethesda, MD 20892; [3]Department of Biochemistry, Faculty of Medicine, University of Toronto, ON, Canada; [4]Departments of Chemistry and Medicinal Chemistry, Purdue University, West Lafayette, IN 47907; [5]National Center for Global Health and Medicine Research Institute, Tokyo 162-8655.

*Correspondence should be addressed to : H.M.

Postal address: Hiroaki Mitsuya, M.D., Ph.D.
Departments of Infectious Diseases and Hematology, Kumamoto University School of Medicine
1-1-1 Honjo, Kumamoto 860-8556, Japan
Phone: (+81) 96-373-5156, Facsimile: (+81) 96-363-5265
e-mail: hm21q@nih.gov

Supplemental Table 1. The identified amino acid substitutions in the protease-encoding region of PI-resistant strains.

Strains	Amino acid substitutions in PR[a]
HIV-$1_{ERS104pre}$ (wild-type)	L63P
HIV-$1_{MDR/B}$	L10I, K14R, L33I, M36I, M46I, F53I, K55R, I62V, L63P, A71V, G73S, V82A, L90M, I93L
HIV-$1_{MDR/C}$	L10I, I15V, K20R, L24I, M36I, M46L, I54V, I62V, L63P, K70Q, V82A, L89M
HIV-$1_{MDR/G}$	L10I, V11I, T12E, I15V, L19I, R41K, M46L, L63P, A71T, V82A, L90M
HIV-$1_{MDR/TM}$	L10I, K14R, R41K, M46L, I54V, L63P, A71V, V82A, L90M, I93L
HIV-$1_{MDRmix\ DRV^{R}20P}$	L10I, I15V, K20R, L24I, V32I, M36I, M46L, L63P, A71T, V82A, L89M
HIV-$1_{ATV^{R}5\mu M}$	L23I, E34Q, K43I, M46I, I50L, G51A, L63P, A71V, V82A, T91A
HIV-$1_{LPV^{R}5\mu M}$	L10F, M46I, I54V, V82A
HIV-$1_{APV^{R}5\mu M}$	L10F, V32I, M46I, I54M, A71V, I84V

[a]The amino acid substitutions identified in the protease-encoding region compared to the consensus type B sequence cited from the Los Alamos database in HIV-$1_{ERS104pre}$, HIV-$1_{MDR/B}$, HIV-$1_{MDR/C}$, HIV-$1_{MDR/G}$, HIV-$1_{MDR/TM}$, HIV-$1_{MDRmix\ DRV^{R}20P}$ were shown. The amino acid substitutions identified in the protease-encoding region compared to the wild-type HIV-1_{NL4-3} in HIV-$1_{ATV^{R}5\mu M}$, HIV-$1_{LPV^{R}5\mu M}$, or HIV-$1_{APV^{R}5\mu M}$ were also shown.

Supplemental Table 2. Antiviral activity of GRL-09510 against X4 tropic subtype-A strain, dual tropic subtype B strain, or R5 tropic subtype B strain in PHA-PBMCs.

Virus[a]	EC_{50} (μM)[b]			
	GRL-09510	APV	ATV	DRV
HIV-$1_{ERS104pre}$ (Wild-type, X4)	0.0035 ± 0.0001	0.039 ± 0.005	0.0031 ± 0.0006	0.0044 ± 0.0009
HIV-1_{ME46} (subtype B, X4/R5)	0.0012 ± 0.0002	0.022 ± 0.04	0.0042 ± 0.0005	0.0023 ± 0.0004
HIV-$1_{92UG029}$ (subtype A, X4)	0.0027 ± 0.0001	0.041 ± 0.002	0.006 ± 0.003	0.0055 ± 0.0001
HIV-1_{BaL} (subtype B, R5)	0.0031 ± 0.0004	0.029 ± 0.002	0.002 ± 0.001	0.0018 ± 0.0001

[a]X4, R5, and X4/R5 denote X4-tropic HIV-1 strain, R5-tropic HIV-1 strain, and dual-tropic HIV-1 strain, respectively.

[b]The EC_{50} (50 % effective concentration) values were determined by using PHA-PBMC as target cells and the inhibition of p24 Gag protein production by each drug was used as an endpoint. All assays were conducted in duplicate or triplicate, and the data shown represent mean values (± 1 S.D.) derived from the results of two independent experiments. PHA-PBMCs were derived from a single donor in each independent experiment.

Supplemental Table 3. Antiviral activity of GRL-09510 and various PIs against a GRL-09510-selected HIV-1 variant, HIV-$1_{9510}{}^{R}{}_{36P}$.

Virus	EC_{50} (μM)					
	SQV	APV	ATV	LPV	DRV	GRL-9510
HIV-1_{NL4-3}	0.018 ± 0.001	0.030 ± 0.006	0.0043 ± 0.0009	0.050 ± 0.007	0.0045 ± 0.0004	0.0022 ± 0.0009
HIV-$1_{9510}{}^{R}{}_{P36}$	0.041 ± 0.009 (2)	0.27 ± 0.02 (9)	0.024 ± 0.001 (6)	0.40 ± 0.02 (8)	0.041 ± 0.001 (9)	0.024 ± 0.002 (11)

MT-4 cells (10^4) were exposed to 100 $TCID_{50}$ of each HIV-1 isolate and the inhibition of p24 Gag protein production by each drug was used as an endpoint. The numbers in parentheses represent the fold changes of EC_{50} values for HIV-$1_{9510}{}^{R}{}_{36P}$ compared to the EC_{50} values for HIV-1_{NL4-3}. All assays were conducted in duplicate and the data shown represent mean values (± 1 standard deviations) derived from the results of two to three independent experiments.

Supplemental Table 4. Comparison of anti-HIV-1 activity between GRL-09510, -0519, -0739, -04810, -05010, and DRV against selected HIV-1 strains.

	EC_{50} (nM)[b]					
Virus[a]	GRL-09510	GRL-0519*	GRL-0739*	GRL-04810*	GRL-05010*	DRV
HIV-1_{NL4-3}	2.8 ± 0.7	0.5 ± 0.1	3.4 ± 0.9	0.5 ± 0.5	3.7 ± 0.1	4.5 ± 0.4
HIV-$1_{WT/ERS104pre}$	3.5 ± 0.1	0.6 ± 0.2	3.6 ± 0.3	2.3 ± 0.1	2.7 ± 0.3	4.4 ± 0.9
HIV-$1_{MDR/G}$	3.3 ± 1.4 (1)	2.6 ± 1.3 (4)	10 ± 7 (3)	4 ± 1 (2)	4 ± 1 (1)	20 ± 9 (5)
HIV-$1_{APV^R 5\mu M}$	4.8 ± 0.1 (2)	38.0 ± 0.9 (76)	310 ± 20 (91)	430 ± 20 (860)	560 ± 30 (187)	410 ± 10 (93)
HIV-$1_{MDRmixDRV^R 20P}$	25 ± 1 (7)	30.0 ± 9.8 (50)	n.d.	n.d.	n.d.	300 ± 60 (68)

[a]Explanations of each HIV-1 strain is provided in footnotes of Tables 2 and 3. [b]EC_{50} values were determined by MT-4 cells employing p24 assay for HIV-1_{NL4-3} and HIV-$1_{APV^R 5\mu M}$, by PHA-PBMs employing p24 assay for HIV-$1_{WT/ERS104pre}$, HIV-$1_{MDR/G}$, and HIV-$1_{MDRmixDRV^R 20P}$, respectively. The data shown represent mean values derived from the results of two or three independent experiments. PBM were derived from a single donor in each independent experiment. The numbers in parentheses represent the fold changes in the EC_{50}s for HIV-$1_{APV^R 5\mu M}$ compared to the EC_{50}s for HIV-1_{NL4-3}, or the fold changes in the EC_{50}s for HIV-$1_{MDR/G}$ and HIV-$1_{MDRmixDRV^R 20P}$ compared to the EC_{50}s for HIV-$1_{WT/ERS104pre}$, respectively. n.d; not determined. *Antiviral data of GRL-0519, -073, -04810 and -05010 were previously reported by us in references 20, 23, and 22, respectively.

Supplemental Table 5. Primers and PCR condition used for the determination of nucleotide sequences.

Primers	Sequence
LTR F1	5’-GAT GCT ACA TAT AAG CAG CTG C-3’
PR12	5’-CTC GTG ACA AAT TTC TAC TAA TGC-3’
LTR F2	5’-GAG ACT CTG GTA ACT AGA GAT C-3’
KSMA2.1	5’-CCA TCC CGG GCT TTA ATT TTA CTG GTA C-3’
**PCR**	**PCR Condition**
1st PCR	Initial 3 m at 95 °C, 35 cycles of 40 s at 95 °C, 20 s at 55 °C, and 2 m at 72 °C, final 10 m of extension at 72 °C
2nd PCR	Initial 3 m at 95°C, 35 cycles of 30 s at 95 °C, 20 s at 55 °C, and 2 m at 72 °C, final 10 m of extension at 72 °C.

Supplemental Table 6. X-ray diffraction data processing details and structure refinement statistics for the X-ray crystal structure of PR_{WT} in complex with GRL-09510.

	PR_{WT} + GRL-09510
PDB entry	5V4Y
Resolution range (Å)	50.00 - 1.80
Unit cell - a (Å)	62.473
b (Å)	62.473
c (Å)	82.929
α (°)	90.0
β (°)	90.0
γ (°)	120.0
Space group	$P6_122$
Solvent content (%)	54.23
No. of unique reflections	9082
Mean ($I/\sigma(I)$)	26.407 [a](2.088)
[b]R_{merge}	0.080 (0.446)
Data redundancy	2.9 (2.9)
Completeness (%)	96.8 (98.5)
Resolution range (Å)	29.23 - 1.90
No. of reflections used	7,770
[c]R_{cryst}	0.2058
R_{free}	0.2533
No. of protein atoms	755

No. of ligand atoms	41
No. of water molecules	73
Mean temperature factors - protein (Å^2)	31.113
Main chains (Å^2)	28.728
Side chains (Å^2)	33.744
Ligand (Å^2)	22.904
Waters (Å^2)	36.828
RMSD bond lengths (Å)	0.009
RMSD bond angles (Å)	1.114
Ramachandran plot - Most favored (%)	98.97
Additional allowed (%)	1.03
Generously allowed (%)	0
Disallowed (%)	0

[a]Values in parentheses are for the highest resolution shell[b]$R_{merge} = \Sigma |I - \langle I \rangle| / \Sigma I$[c]$R_{cryst} = \Sigma ||F_{obs}| - |F_{calc}|| / \Sigma|F_{obs}|$

Supplemental Figure 1. Locations of amino acid substitutions identified in protease of HIV-1_{NL4-3} selected in the presence of increasing concentrations of GRL-09510.

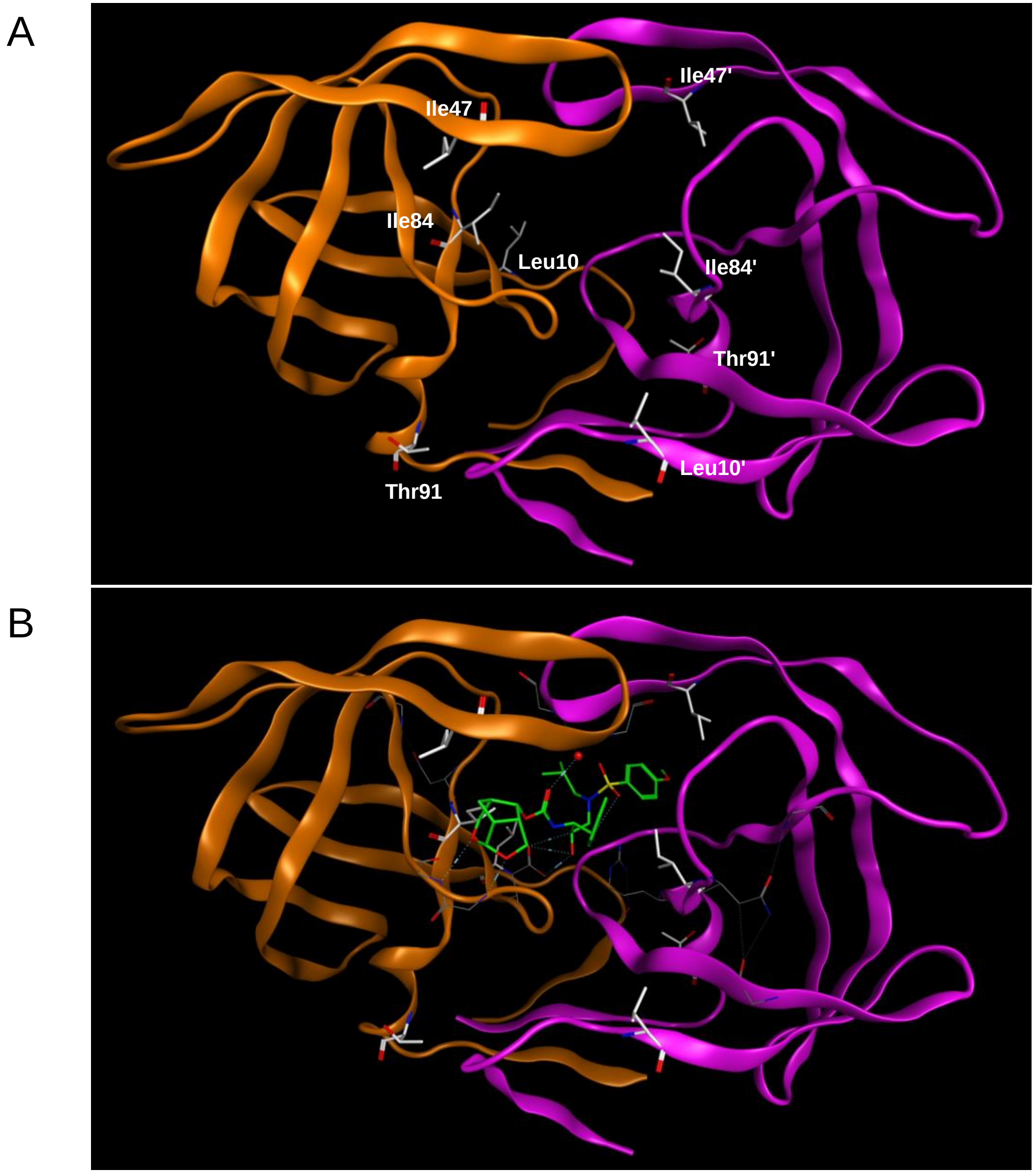

(A) The locations of amino acid substitutions observed in the selection experiment in the PR dimer are illustrated. (B) The locations of amino acid substitutions observed in the selection experiment in the wild-type PR dimer and GRL-09510 (green) complex are illustrated.

Supplemental Figure 2. Structures of GRL-0519, -0739, -04810, and -05010.

GRL-0519

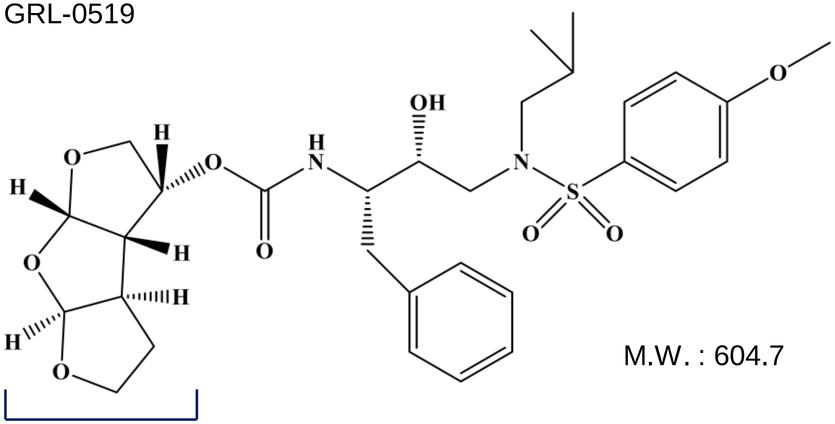

Tris-THF

M.W. : 604.7

GRL-0739

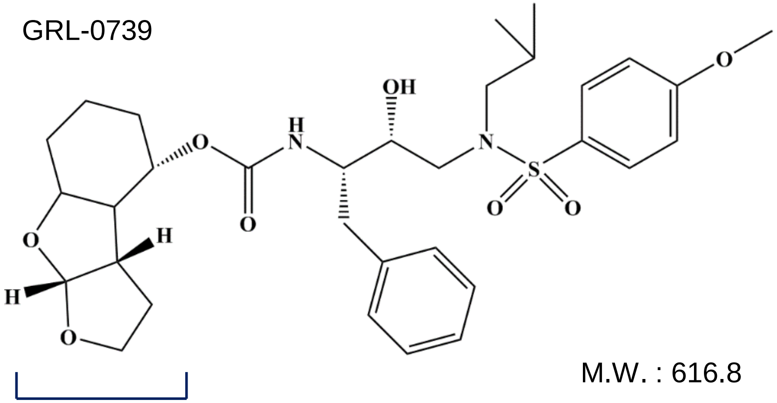

Cyclohexyl-*bis*-THF

M.W. : 616.8

GRL-04810

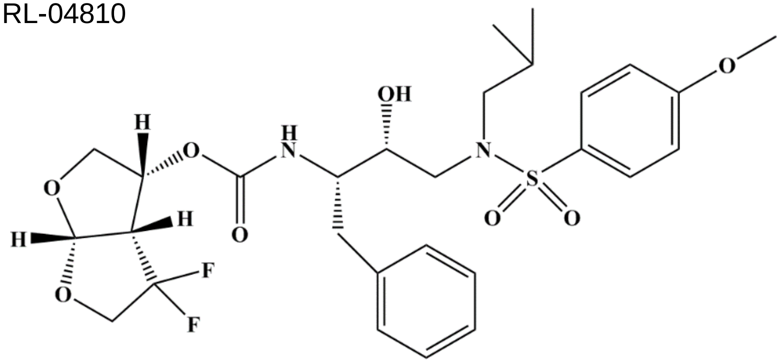

M.W. : 598.7

GRL-05010

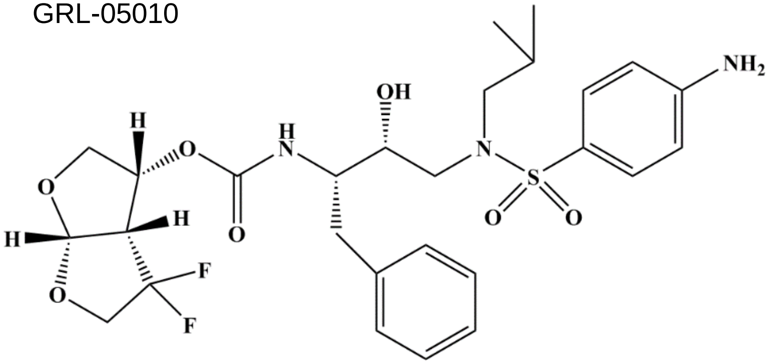

M.W. : 583.7
